# Search Filters for Finding Prognostic and Diagnostic Prediction Studies in Medline to Enhance Systematic Reviews

**DOI:** 10.1371/journal.pone.0032844

**Published:** 2012-02-29

**Authors:** Geert-Jan Geersing, Walter Bouwmeester, Peter Zuithoff, Rene Spijker, Mariska Leeflang, Karel Moons

**Affiliations:** 1 Julius Center for Health Sciences and Primary Care, University Medical Center Utrecht, Utrecht, The Netherlands; 2 Medical Library Academic Medical Center, University of Amsterdam, Amsterdam, The Netherlands; 3 Department of Clinical Epidemiology and Bio-Informatics, Academic Medical Center, University of Amsterdam, Amsterdam, The Netherlands; 4 Dutch Cochrane Center, Academic Medical Center, University of Amsterdam, Amsterdam, The Netherlands; University of Illinois-Chicago, United States of America

## Abstract

**Background:**

The interest in prognostic reviews is increasing, but to properly review existing evidence an accurate search filer for finding prediction research is needed. The aim of this paper was to validate and update two previously introduced search filters for finding prediction research in Medline: the Ingui filter and the Haynes Broad filter.

**Methodology/Principal Findings:**

Based on a hand search of 6 general journals in 2008 we constructed two sets of papers. Set 1 consisted of prediction research papers (n = 71), and set 2 consisted of the remaining papers (n = 1133). Both search filters were validated in two ways, using diagnostic accuracy measures as performance measures. First, we compared studies in set 1 (reference) with studies retrieved by the search strategies as applied in Medline. Second, we compared studies from 4 published systematic reviews (reference) with studies retrieved by the search filter as applied in Medline. Next – using word frequency methods – we constructed an additional search string for finding prediction research. Both search filters were good in identifying clinical prediction models: sensitivity ranged from 0.94 to 1.0 using our hand search as reference, and 0.78 to 0.89 using the systematic reviews as reference. This latter performance measure even increased to around 0.95 (range 0.90 to 0.97) when either search filter was combined with the additional string that we developed. Retrieval rate of explorative prediction research was poor, both using our hand search or our systematic review as reference, and even combined with our additional search string: sensitivity ranged from 0.44 to 0.85.

**Conclusions/Significance:**

Explorative prediction research is difficult to find in Medline, using any of the currently available search filters. Yet, application of either the Ingui filter or the Haynes broad filter results in a very low number missed clinical prediction model studies.

## Introduction

Clinical prediction research aims to facilitate in individual risk prediction of absolute probabilities (or risks) for either the presence of a certain disease (diagnostic research) or the occurrence of a future outcome (prognostic research) [1], [2], [3]. Various studies have shown that tools to enable such individual risk prediction may enable a more cost-effective use of healthcare resources, a better classification of patients in risk groups than physicians' judgement only, and minimizes patient burden [4]–[10]. Not surprisingly, prediction research has been a topic of increasing interest over the last few decades in the medical literature [11], [12], [13].

Introduction of evidence from prediction research in daily clinical practice is hampered for several reasons that may be specific for this type of research. First, the number of potential new predictors – such as biomarkers and genetic loci – as well as new prediction models increases almost on a daily basis [14]–[17]. In addition – much more than in therapeutic research – new studies often find conflicting results on potential predictors, possibly due to relatively small sample sizes [18]. Hence, there is an urgent need for systematic reviews on prediction research, including meta-analyses, where possible [19], [20], [21].

As a first step for such reviews, finding relevant studies in electronic databases such as Medline is important. For that purpose, several generic search filters have been developed that can be used to find relevant prediction research studies [22], [23], [24]. Generic search filters for prediction research commonly combine epidemiological terms related to prediction research (see table 1). These filters than in turn are combined with disease specific terms. Accordingly, they are used to find prediction research in electronic databases on a specific disease. Just as it is widely acknowledged that clinical prediction models often perform less when tested in a new set of patients, search filters may fail in identifying all relevant studies when it is used in a different set of papers than the set with which it was developed. Therefore, the objective of this study is to validate and – if needed – update existing generic search filters for finding prediction research. We aimed to find an optimal generic search filter for systematic review purposes, meaning that the search filter would ideally find all relevant papers on a specific topic. In addition, we aimed to give recommendations for future researchers that will embark on a systematic literature search for review purposes of prediction research.

**Table 1:** Search strategies for finding prediction research in Medline.

Filter	Search terms included in the filter*	Sensitivity# (95% CI)	Specificity# (95% CI)
Ingui filter	(Validat$ OR Predict$.ti. OR Rule$) OR (Predict$ AND (Outcome$ OR Risk$ OR Model$)) OR ((History OR Variable$ OR Criteria OR Scor$ OR Characteristic$ OR Finding$ OR Factor$) AND (Predict$ OR Model$ OR Decision$ OR Identif$ OR Prognos$)) OR (Decision$ AND (Model$ OR Clinical$ OR Logistic Models/)) OR (Prognostic AND (History OR Variable$ OR Criteria OR Scor$ OR Characteristic$ OR Finding$ OR Factor$ OR Model$))	0.98 (0.92–1.0)	0.86 (0.85–0.87)
Haynes broad filter	(Predict*[tiab] OR Predictive value of tests[mh] OR Scor*[tiab] OR Observ*[tiab] OR Observer variation[mh])	0.96	0.79

*Using the Pubmed interface for MEDLINE.

# Sensitivity and specificity as reported by Ingui and Haynes in their original publication; CI = confidence interval, for the Haynes broad filter no confidence intervals were given in the original publication.

## Results

### Validation of the existing search filters

#### Hand searched papers

We manually reviewed 1204 papers that were published in 2008 in our set of 6 general medical journals. In total, 71 papers were classified as prediction research: 51 “predictor finding studies”, 17 “clinical prediction model studies (development, validation or combination)”, and 3 “impact studies”. Overall, both the Ingui filter and the broad Haynes filter identified 52 of these 71 studies (sensitivity of 0.73; Table 2). Clinical prediction model studies were almost always correctly retrieved by both search strategies: all 17 by the Ingui filter and 16/17 by the broad Haynes filter (sensitivity 1.0 and 0.94 respectively). For predictor finding studies both filters identified 34 out of 51 (sensitivity 0.67). Our hand search only yielded 3 “prediction model impact studies”, of these 1 one was identified by the Ingui filter and 2 by the broad Haynes filter.

**Table 2:** Accuracy of search filters for finding predictor finding studies and clinical prediction model studies.

	Hand search		Meta-analysis 1		Meta-analysis 2	
	Sensitivity (95% CI)	Specificity (95% CI)	Sensitivity (95% CI)	NNR	Sensitivity (95% CI)	NNR
**Ingui filter**						
Overall	0.73 (0.62–0.82)	0.77 (0.74–0.79)	N.A.	N.A.	N.A.	N.A.
PF studies	0.67 (0.53–0.78)	0.76 (0.73–0.78)	0.44 (0.34–0.56)	374	N.A.	N.A.
CPM studies	1.0 (0.82–1.0)	0.75 (0.72–0.77)	0.83 (0.66–0.92)	54	0.78 (0.55–0.91)	103
**Haynes filter**						
Overall	0.73 (0.62–0.82)	0.68 (0.65–0.71)	N.A.	N.A.	N.A.	N.A.
PF studies	0.67 (0.53–0.78)	0.67 (0.64–0.70)	0.85 (0.75–0.91)	907	N.A.	N.A.
CPM studies	0.94 (0.73–0.99)	0.67 (0.64–0.70)	0.83 (0.66–0.92)	208	0.89 (0.67–0.97)	364

CI = confidence interval; PF = predictor finding; CDR = clinical prediction model; NNR = number needed to read; N.A. = not applicable.

Meta-analysis 1 for PF studies = Systematic review of prognostic models in patients with acute stroke – C Counsell et.al. Cerebrovasc Dis 2001; 12:159–70.

Meta-analysis 1 for CDR studies = Clinical prediction rules for pulmonary embolism: a systematic review and meta-analysis – E Ceriani et.al. JTH 2010;8:957–70.

Meta-analysis 2 for CDR studies = Accuracy and quality of clinical decision rules for syncope in the emergency department: a systematic review and meta-analysis – LA Serrano et.al. Ann of Emerg Med 2010;56:362–73.

#### Existing meta-analyses

We additionally validated both filters in existing systematic reviews. For “predictor finding studies” we used a systematic review on prognostic factors in patients with acute stroke [25]. This systematic review included 70 Medline indexed papers. The Ingui filter yielded a sensitivity of 0.44, and the Haynes a sensitivity of 0.85, with an efficiency or number needed to read of NNR = 907 (Table 2).

We used two existing systematic reviews for clinical prediction model studies [26], [27]: one on prediction models for patients with suspected pulmonary embolism (including 29 Medline indexed papers), and one on prediction models in patients with syncope presenting at an emergency department (including 18 Medline indexed papers). Similar as in our hand search, both filters correctly retrieved most of these papers (sensitivity ranged between 0.78 [prediction models for pulmonary embolism using the Ingui filter], and 0.89 [prediction models for pulmonary embolism using the Haynes filter]). Efficiency was highest for the Ingui filter: NNR of 54 and 103 for the Ingui filter, as compared to a NNR of 208 and 364 for the broad Haynes filter.

For validating both filters on existing reviews on prediction model impact studies we used a landmark paper on this topic by Reilly and Evans that described 5 impact papers [13]. The Ingui filter missed 1 of the 5, and the Haynes filter 2 (Table 3).

**Table 3:** Accuracy of search filters for finding impact studies.

	Hand search (n = 3)	Meta-analysis (n = 5)
**Ingui filter**		
# of studies retrieved by filter	1	4
# of studies not retrieved by filter	2	1
**Haynes filter**		
# of studies retrieved by filter	2	3
# of studies not retrieved by filter	1	2

# = number.

### Optimising the search filters

Our updating process yielded an additional search string (see Table 4).

**Table 4:** Updated search string for finding prediction research.

“Stratification” OR “ROC Curve”[Mesh] OR “Discrimination” OR “Discriminate” OR “c-statistic” OR “c statistic” OR “Area under the curve” OR “AUC” OR “Calibration” OR “Indices” OR “Algorithm” OR “Multivariable”

“Stratification” OR “ROC Curve”[Mesh] OR “Discrimination” OR “Discriminate” OR “c-statistic” OR “c statistic” OR “Area under the curve” OR “AUC” OR “Calibration” OR “Indices” OR “Algorithm” OR “Multivariable”. We validated this search string in the previously described systematic reviews [13], [25], [26], [27] by combining it with the Ingui filter or the Haynes broad filter using the Boolean operator “OR”.

For the predictor finding studies review, a small increase in the sensitivity was observed for both the Ingui filter and the Haynes broad filter, as compared to the sensitivity of both original filters alone (Table 5). For the prediction model studies review, notably the Ingui filter combined with our additional search string resulted in an increase in sensitivity from around 0.80 (table 2) to around 0.95 (range 0.94 to 0.97; table 6).

**Table 5:** Updated search strings for predictor finding studies.

	Meta-analysis search	
Ingui filters	Sensitivity (95% CI)	NNR
“Ingui filter” OR “update”	0.47 (0.36–0.59)	569

Haynes filter		
“Haynes broad filter” OR “update”	0.84 (0.74–0.91)	1010

CI = confidence interval; NNR = number needed to read.

Updated search string = “Stratification” OR “ROC Curve”[Mesh] OR “Discrimination” OR “Discriminate” OR “c-statistic” OR “c statistic” OR “Area under the curve” OR “AUC” OR “Calibration” OR “Indices” OR “Algorithm” OR “Multivariable”.

Meta-analysis for predictor finding studies = Systematic review of prognostic models in patients with acute stroke – C Counsell et.al. Cerebrovasc Dis 2001; 12:159–70.

**Table 6:** Updated search strings for finding clinical prediction models studies.

	Meta-analysis 1		Meta-analysis 2	
Ingui filters	Sensitivity (95% CI)	NNR	Sensitivity (95% CI)	NNR
“Ingui filter” OR “update”	0.97 (0.83–0.99)	68	0.94 (0.74–0.99)	125

Haynes filter				
“Haynes broad filter” OR “update”	0.90 (0.74–0.96)	208	0.89 (0.67–0.97)	395

CI = confidence interval; NNR = number needed to read.

Updated search string = “Stratification” OR “ROC Curve”[Mesh] OR “Discrimination” OR “Discriminate” OR “c-statistic” OR “c statistic” OR “Area under the curve” OR “AUC” OR “Calibration” OR “Indices” OR “Algorithm” OR “Multivariable”.

Meta-analysis 1 for clinical prediction models studies = Clinical prediction rules for pulmonary embolism: a systematic review and meta-analysis – E Ceriani et.al. JTH 2010;8:957–70.

Meta-analysis 2 for clinical prediction models studies = Accuracy and quality of clinical decision rules for syncope in the emergency department: a systematic review and meta-analysis – LA Serrano et.al. Ann of Emerg Med 2010;56:362–73.

## Discussion

We validated and updated two existing generic search filters for finding (various types of) prediction research in Medline. Our validation and updating process was based on both a hand search in 6 general journals in 2008 and additional validation in 4 published systematic reviews. Studies on finding relevant predictors for a certain outcome can be quite problematic: all existing as well as the updated generic search filters showed a sensitivity ranging from 40% to 80%, and were also hampered by a low efficiency (this is, many studies have to be screened to find one relevant study). Studies on clinical prediction models can much better be traced in Medline: more than 90% can be retrieved when combining either the Haynes broad filter or the Ingui filter with the additional search string developed in this study. Finally, prediction model impact studies are still rare in the medical literature. Our hand search only yielded 3 of such studies in 2008; a previous review in the same journals from 2000 to 2003 also included only 5 papers. We believe that a formal search string to find such prediction studies can therefore currently not be properly developed. Both existing filters are not very good in identifying these studies.

### Strengths and limitations

A strength of our study is that our validation process was based not only on a full and comprehensive hand search of 6 major journals, but also included an additional validation in 4 systematic reviews on prediction research. However, for full appreciation of our results some issues deserve attention.

First, despite the full comprehensive hand search of 6 journals, the number of retrieved prediction studies was still relatively low, in particular for the impact studies. As a consequence, the generalizability of our results for impact studies may be limited. Although partly caused by the fact that we only hand searched journals in 2008, this problem currently is hard to overcome. Impact studies are still rare, even though researchers, journal editors and clinicians have stressed the need for it [7], [13], [28]. Second, our definition of the different predictor study types can be somewhat arbitrary. However, this definition was based on a previous series on prediction research as well as on other methodological discussions and guidelines on prediction research [15], [29], [30]. Third, our hand search was based on 6 general medical journals. An alternative approach could be to (also) include more specialist journals. We explicitly chose the 6 general medical journals, as they commonly publish on prediction research on a wide spectrum of different diseases, thereby increasing the generalizability of our results. Fourth, similar to Ingui, we used automated word count frequency methods (i.e. PubReminer and Termine – see “methods” section) to develop our additional search string. A different approach – for example a manual word count frequency method or consulting experts on prediction research on relevant terms for developing an additional search string – inherently would result in different search terms. Yet, we believe that our method is more transparent and reproducible than both alternative approaches. Thereby, it allows for updating the search string in the future, e.g. when more “impact studies” have become available. Finally, we did not formally assess the quality of the searches of the systematic reviews needed for the additional validation part of our validation analysis. Yet, the number of included studies in the respective systematic reviews was in our view large enough to enable a formal validation process of our included search filters.

### Implications and recommendations for future researchers

Prediction research is abundant in the medical literature [15], [17], [30] and new papers on novel biomarkers, new clinical prediction models or risk schemes on both diagnostic and prognostic clinical problems are increasing on an almost daily basis. Usually, for clinicians working in daily practice this overwhelming amount of prediction studies is not very helpful. Hence, there is an urgent need for systematic reviews on prediction research, including meta-analyses where possible. Yet, finding relevant studies can be quite difficult as prediction studies are not indexed as such in Medline, as is for example the case for randomized trials [31]. Therefore, researchers that embark on a systematic review have to use extensive search filters to identify relevant studies.

Based on our study, such researchers can apply the following recommendations. If the aim is to find explorative “predictor finding studies” no generic search filter (combined with subject matter terms) can find all relevant studies. Therefore, if the interest of the systematic review is focused on only one or two potential predictors or (bio) markers, it can be desirable not to use any generic search filter. Instead, the Medline search should be largely focused on combining the terms for the potential predictor or marker combined with the disease or outcome at interest (for example, combining D-dimer test with venous thrombo-embolism [32]). If one is interested, however, in a review on the several (partly unknown) predictors or markers or models for a particular disease or outcome, the use of generic search strategies as validated and updated in this study is almost inevitable. A first step here could be combining the Ingui filter or the Haynes broad filter – plus the additional search string described in this study – and combined with the disease and/or outcome at interest. The so retrieved studies can then be used for cross-reference checking. Cross-reference checking implies that all reference lists of retrieved studies are checked for any additional studies that were not yet retrieved by the MEDLINE search. Additionally, authors of these studies then can be contacted in order to identify more studies.

For systematic reviews on prediction model studies, there are good (generic) search filters to find almost all relevant studies. Both the Haynes broad filter and the Ingui filter have high retrieval rates for finding such studies in Medline, with the best performance for the Ingui filter plus the additional search string that we developed in our study. Therefore, researchers can feel quite confident that combining such a search filter with the disease or outcome at interest finds most – if not all – available prediction model studies. Preferably they should double-check their retrieved study references with a known expert in the field.

Unfortunately, no valid recommendations can currently be drawn for finding “impact studies”. The generic search strings that currently are available do not seem to find all relevant studies. This is largely due to the fact that such studies are still quite rare in the medical literature.

### Conclusion

Combining search filters with the disease of interest enables an accurate identification of studies on clinical prediction models. If the aim of the systematic review is (also) to find the more explorative studies on finding predictors or finding studies on the impact of clinical prediction models, the Medline search should never be based on only a search filter for prediction research combined with the disease of interest.

## Materials and Methods

### Definition of prediction research

Building on previous guidelines [12], [13], [29], [33], we here also distinguish three types of prediction research (for both diagnostic and prognostic prediction research). First “predictor finding studies”, which aim to discover or explore which predictors or variables out of a number of candidate predictors, independently contribute to (are associated with) the prediction of an outcome. Second “clinical prediction model studies”, which aim to develop and/or (externally) validate a multivariable prediction model (which combine multiple predictors to a single model or tool) for use in medical practice to guide patient management. Such studies may aim to identify the most important predictors, assign the (mutually adjusted) weights per predictor in a multivariable analysis, to develop a final multivariable prediction model, and to validate its predictive accuracy in other subjects than in whom the model was developed. A key aspect in these ‘prediction model development and validation studies’ is to estimate the model's predictive performance (e.g. calibration and discrimination statistics) in a specific cohort of subjects. The third type of prediction studies may be the “impact studies”, which aim to quantify the effect or impact of using a prognostic or diagnostic prediction model on physicians' behaviour, patient outcome or cost-effectiveness of care relative to not using the model or usual care. Here not so much the model's predictive performance is studied in a single cohort, but rather the effects of its use as compared to not using the model, on clinical decision-making and subsequent patient outcomes. Hence, a comparative design is used for such studies.

### Search filters under study

For this study we evaluated two existing search filters for finding prediction research in the medical literature: a search filter as proposed by Ingui and co-workers [23], and a search filter that was developed by the Hedges team [24].

In 2001 Ingui and co-workers developed several search filters for finding multivariable clinical prediction models [23]. Their search filters were based on 119 articles on clinical prediction models manually retrieved from 6 general medical journals published between 1991 and 1998. This set was subsequently split into a derivation set and a validation set. For our study, we included their search filter that yielded the highest combination of sensitivity and specificity.

In the early 90 s, the Hedges team developed – based on a set of 10 journals – search filters for finding four types of articles in Medline (therapy, diagnosis, prognosis and causation). These filters were updated using ‘methodologically sound’ papers manually selected from 161 Medline-indexed journals published in 2000. The authors identified 91 articles on clinical prediction models. A comprehensive set of search terms (based on interviews with known experts) were subsequently tested on these 91 articles [24]. We validated this search filter that is also made available in the ‘Clinical Queries’ section of Pubmed, and that is referred to as the Haynes broad filter.

Both search filters are summarized in table 1.

### Validation of the search filters

Using a similar approach as Ingui and the Hedges team, we first constructed a database of prediction research studies, including all three above types of medical prediction research. Our database was constructed using a full manual search of all articles published in 6 general medical journals in 2008: Annals of Internal Medicine, BMJ, JAMA, Lancet, New England Journal of Medicine, and PlosMedicine. This manually compiled document of our hand search is available upon request, by contacting the corresponding author (GJG).

Two authors (WB and KGMM) categorised the retrieved papers in two groups: prediction research studies (set 1; either predictor finding, prediction model development/validation, or model impact studies) versus non-prediction research studies (set 2). Studies in set 1 could either be found (true positives, TP) or missed by the search filter under study (false negatives, FN); similarly, studies in set 2 could either falsely be identified as prediction research (false positives, FP) or correctly identified as non-prediction research (true negatives, TN).

The Ingui search filter and the Haynes broad filter were validated for finding all prediction research types combined, as well as for ‘predictor finding studies’, ‘clinical prediction model studies’, and ‘impact studies’ separately. In accordance with previous studies, we used the diagnostic accuracy measures sensitivity ( = TP/[TP + FN]) and specificity ( = TN/[TN + FP]) as performance measures for these search strategies [22], [34].

As our aim was to find optimal search filters for systematic review purposes, we additionally validated both the Ingui search filter and the Haynes broad filter in four existing systematic reviews: one review on ‘predictor finding studies’ [25], two reviews on ‘clinical prediction models’ [26], [27] and one review on ‘impact studies’ [13]. We assumed that the retrieved articles in these reviews were based on a complete and thorough search of the literature as well as contacts with experts in the field, and regarded the set of articles used in these reviews as a reference. We combined the Ingui search filter or the Haynes broad filter with the same subject matter (e.g. disease, outcome or predictor) related terms as used in the respective meta-analysis. Accordingly, articles in the dataset of the respective meta-analysis could either also be found by this search (true positive, TP) or be missed (false negative, FN). Subsequently, sensitivity was calculated. As these datasets consists only of relevant articles and not of irrelevant articles, we could not calculate the specificity of the Ingui and Haynes filter in the four meta-analyses. Instead, we calculated the ‘number needed to read’ (NNR) as a performance measure [35]. NNR is calculated by dividing the total number of articles found in Medline with the number of true positives. This performance measure can be interpreted as the total number of articles that researchers have to screen before finding one relevant paper, reflecting the efficiency of the search filter.

### Updating of the search filters

Two methods were employed to evaluate whether the two search filters could be improved, both aimed at identifying unique discriminating search terms. As described above, all scientific articles from 2008 appearing in 6 general medical journals were screened and divided into two sets: set 1 containing all relevant prediction research studies and set 2 containing all non-relevant articles. First – using PubReminer (http://bioinfo.amc.uva.nl/human-genetics/pubreminer/) – a frequency analysis of both sets was performed to determine the most frequently used text words and Mesh-terms [36]. In short, PubReminer submits a user query to PubMed and retrieves the Medline abstracts for all citations matching the query. The abstracts were then split into separate words (merging related terms) and used for the generation of frequency tables. These frequency lists are then reported to the user in interactive tables. Next to Pubreminer, we used the web-based service TerMine [37]. TerMine was used to compare nested multi-word terms within context between both sets of our database (http://www.nactem.ac.uk/software/termine/).

Both methods were used to identify an additional search string composed of the most discriminatory search terms between prediction (set 1) and non-prediction (set 2) research. As this search string was developed on our complete database of prediction research studies, validating it in this same database would yield too optimistic results. Therefore, we only validated it in the above-mentioned existing meta-analyses.
