# Correction: Search Filters for Finding Prognostic and Diagnostic Prediction Studies in Medline to Enhance Systematic Reviews

**DOI:** 10.1371/annotation/96bdb520-d704-45f0-a143-43a48552952e

**Published:** 2012-07-10

**Authors:** Geert-Jan Geersing, Walter Bouwmeester, Peter Zuithoff, Rene Spijker, Mariska Leeflang, Karel Moons

There was an error in the sixth author's name. Karel G.M. Moons is correct.

